# Primary versus secondary antiemetic prophylaxis with NK1 receptor antagonists in patients affected by gastrointestinal malignancies and treated with a doublet or triplet combination regimen including oxaliplatin and/or irinotecan plus fluoropyrimidines: A propensity score matched analysis

**DOI:** 10.3389/fonc.2022.935826

**Published:** 2022-08-12

**Authors:** Alessandro Parisi, Riccardo Giampieri, Alex Mammarella, Cristiano Felicetti, Lisa Salvatore, Maria Bensi, Maria Grazia Maratta, Antonia Strippoli, Roberto Filippi, Maria Antonietta Satolli, Angelica Petrillo, Bruno Daniele, Michele De Tursi, Pietro Di Marino, Guido Giordano, Matteo Landriscina, Pasquale Vitale, Ina Valeria Zurlo, Emanuela Dell’Aquila, Silverio Tomao, Ilaria Depetris, Francesca Romana Di Pietro, Federica Zoratto, Davide Ciardiello, Maria Vittoria Pensieri, Ornella Garrone, Barbara Galassi, Claudio Ferri, Rossana Berardi, Michele Ghidini

**Affiliations:** ^1^ Clinica Oncologica e Centro Regionale di Genetica Oncologica, Università Politecnica delle Marche, AOU Ospedali Riuniti-Ancona, Ancona, Italy; ^2^ Department of Life, Health and Environmental Sciences, University of L’Aquila, L’Aquila, Italy; ^3^ Università Cattolica del Sacro Cuore, Rome, Italy; ^4^ Medical Oncology, Comprehensive Cancer Center, Fondazione Policlinico Universitario Agostino Gemelli, IRCCS, Rome, Italy; ^5^ Department of Oncology, University of Turin, Torino, Italy; ^6^ S.C Oncologia Medica 1, Centro Oncologico Ematologico Subalpino (COES), Azienda Ospedaliera Universitaria Città della Salute e della Scienza di Torino, Torino, Italy; ^7^ Medical Oncology Unit, Ospedale del Mare, Naples, Italy; ^8^ Department of Medical, Oral and Biotechnological Sciences and Center for Advance Studies and Technology (CAST), G. D’Annunzio University, Chieti, Italy; ^9^ Clinical Oncology Unit, S.S. Annunziata Hospital, Chieti, Italy; ^10^ Medical Oncology Unit, Department of Medical and Surgical Sciences, University of Foggia, Foggia, Italy; ^11^ Medical Oncology Unit, Santa Chiara Hospital, Trento, Italy; ^12^ Medical Oncology, “Vito Fazzi” Hospital, Lecce, Italy; ^13^ Medical Oncology 1, IRCCS Regina Elena National Cancer Institute, Rome, Italy; ^14^ Department of Radiological, Oncological and Anatomo-Pathological Sciences, Medical Oncology Unit A, Policlinico Umberto I, ‘Sapienza’ University of Rome, Rome, Italy; ^15^ Medical Oncology, ASL TO4, Ospedale Civile di Ivrea, Turin, Italy; ^16^ Istituto Dermopatico dell’Immacolata (IDI), IRCCS, Rome, Italy; ^17^ Medical Oncology, Santa Maria Goretti Hospital, Latina, Italy; ^18^ Oncology Unit, Casa Sollievo della Sofferenza Hospital, San Giovanni Rotondo, Italy; ^19^ Oncology Unit, Department of Precision Medicine, Università degli Studi della Campania “Luigi Vanvitelli”, Naples, Italy; ^20^ Medical Oncology, St. Salvatore Hospital, L’Aquila, Italy; ^21^ Medical Oncology Unit, Fondazione IRCCS Ca’ Granda Ospedale Maggiore Policlinico, Milano, Italy

**Keywords:** gastrointestinal cancers, FOLFOX, FOLFIRI, FOLFOXIRI, FLOT, netupitant/palonosetron, aprepitant, emesis

## Abstract

**Aim:**

The aim of the current study is to investigate the impact of primary compared to secondary chemotherapy-induced nausea and vomiting (CINV) prophylaxis with NK1 receptor antagonists (NK1-RA) in patients affected by gastrointestinal malignancies and treated with oxaliplatin- and/or irinotecan-based doublet or triplet regimens.

**Study design and methods:**

Clinical data of patients affected by gastrointestinal malignancies, treated with an oxaliplatin and/or irinotecan-based doublet or triplet regimen as neo/adjuvant or advanced-line treatment, and who received NK1-RA as primary (from the first cycle of treatment) or secondary (after the onset of CINV with a previous regimen with 5HT3-RA and dexamethasone) prophylaxis for CINV, were retrospectively collected in an observational study involving 16 Italian centers. A propensity score matching was performed by taking into account the following stratification factors: sex (male vs. female), age (< vs. ≥70 years old), overweight (body mass index, BMI < vs. ≥25), underweight (BMI < vs. ≥19), disease spread (early vs. advanced/metastatic), tumor type (esophagogastric cancer vs. the rest, hepatobiliary tumor vs. the rest, colorectal cancer vs. the rest), type of NK1-RA used as primary/secondary prophylaxis (netupitant-palonosetron vs. fosaprepitant/aprepitant), concomitant use of opioids (yes vs. no), concomitant use of antidepressant/antipsychotic drugs (yes vs. no), Eastern Cooperative Oncology Group (ECOG) performance status at the start of NK1-RA treatment (0 vs. 1–2), and intensity of chemotherapy regimen (doublet vs. triplet).

**Results:**

Among 409 patients included from January 2015 to January 2022 and eligible for analysis, 284 (69%) and 125 (31%) were treated with NK1-RA as primary and secondary antiemetic prophylaxis, respectively. After matching, primary NK1-RA use was not associated with higher rates of protection from emesis regardless the emesis phase (acute phase, p = 0.34; delayed phase, p = 0.14; overall phase, p = 0.80). On the other hand, a lower rate of relevant nausea (p = 0.02) and need for rescue antiemetic therapy (p = 0.000007) in the overall phase was found in primary NK1-RA users. Furthermore, a higher rate of both complete antiemetic response (p = 0.00001) and complete antiemetic protection (p = 0.00007) in the overall phase was more frequently observed in primary NK1-RA users. Finally, chemotherapy delays (p = 0.000009) and chemotherapy dose reductions (p = 0.0000006) were less frequently observed in primary NK1-RA users.

**Conclusion:**

In patients affected by gastrointestinal malignancies, a primary CINV prophylaxis with NK1-RA, 5HT3-RA, and dexamethasone might be appropriate, particularly in those situations at higher risk of emesis and in which it is important to avoid dose delays and/or dose reductions, keeping a proper dose intensity of chemotherapy drugs.

## Introduction

Despite the progress achieved in the last years in the supportive care of cancer patients, chemotherapy-induced nausea and vomiting (CINV) remains one of the most limiting chemotherapy-related adverse events. According to national and international guidelines, irinotecan and oxaliplatin are included in the group of moderately emetogenic chemotherapy (MEC), including antineoplastic agents with an extremely broad risk of CINV, ranging from 30% to 90% if no prophylactic antiemetics are adopted (1–3). Because of this wide range, it is difficult to give a recommendation for antiemetic prophylaxis which could be appropriate for all the drugs included in the category. Moreover, this classification does not take into account the emetogenic risk of more drugs administered concomitantly. Indeed, the systemic treatment of the great majority of gastrointestinal malignancies includes the use of irinotecan and oxaliplatin, more frequently combined with fluoropyrimidines, monoclonal antibodies and/or each other, according to primary tumor type, natural course of the disease, and patients’ characteristics. In this respect, the association of chemotherapy agents in doublet or triplet regimens, together with patients (clinical performance status, age, comorbidities, concomitant drugs as opioids and antidepressant agents, electrolytic disorders, and constipation) and disease (peritoneal carcinomatosis with intestinal occlusion and brain metastases) characteristics, might increase the emetogenic risk (1, 4–6). To date, in the MEC category, adding a NK1 receptor antagonist {NK1-RA, i.e., netupitant [in association with palonosetron or netupitant–palonosetron (NEPA)], aprepitant, or fosaprepitant} to an antiemetic regimen with dexamethasone and (for aprepitant and fosaprepitant) a 5-HT3 receptor antagonist (5-HT3-RA) is recommended in carboplatin-treated patients (7), whereas contrasting results about the role of NK1-RAs have been shown in oxaliplatin-treated patients (8, 9), and no formal clinical trials investigated the role of NK1-RAs in patients treated with irinotecan or with intensive regimens including irinotecan and/or oxaliplatin, such as FOLFOXIRI, FOLFIRINOX (associations of irinotecan, oxaliplatin, and 5-fluorouracil), and FLOT (association of oxaliplatin, docetaxel, and 5-fluorouracil) (10–16). Ultimately, data from a real-life setting are lacking.

Drawing from these considerations, we designed an observational, multicenter study to assess the impact of primary compared to secondary CINV prophylaxis treatment with NK1-RA, 5HT3-RA, and dexamethasone in a population of patients affected by gastrointestinal malignancies and treated with oxaliplatin- and irinotecan-based doublet or triplet regimens in clinical practice.

## Materials and methods

### Patient eligibility

This retrospective analysis evaluated consecutive patients affected by gastrointestinal malignancies and treated at 16 Italian Oncology Units (**Supplementary File 1**), from January 2015 to January 2022.

Eligibility criteria were the following: age ≥18 years; histologically confirmed diagnosis of gastrointestinal (gastroesophageal, biliopancreatic, and colorectal) cancer; availability of clinical data concerning patient and disease characteristics; having received at least one cycle of antineoplastic treatment with an oxaliplatin- or irinotecan-based doublet (FOLFOX, XELOX, or FOLFIRI) or triplet (FOLFOXIRI, FOLFIRINOX, FLOT, or variants) regimen; and having received at least one dose of NK1-RA in association with 5HT3-RA and dexamethasone according to local clinical practice, as primary (from the first cycle of chemotherapy) or secondary (after the onset of CINV with the previous antiemetic regimen) CINV prophylaxis. All patients in the secondary prophylaxis cohort should have received a previous antiemetic treatment with 5HT3-RA plus dexamethasone.

### Study design

The main aim of the study was to assess the effectiveness of a triplet treatment with NK1-RA, 5HT3-RA, and dexamethasone as primary compared to secondary prophylaxis for CINV. The measured effective clinical outcomes were the following: protection from acute phase emesis, defined as the lack of acute (within 24 h from the cycle of chemotherapy) vomiting; protection from delayed phase emesis, defined as the lack of delayed (after 24 h from the cycle of chemotherapy) vomiting; protection from overall phase emesis, defined as the sum of both protection from acute and delayed phase emesis; need for rescue therapy for nausea/vomiting during the overall phase; complete response (CR), defined as no acute/delayed phase emesis and no need for rescue therapy for nausea/vomiting during the overall phase; protection from relevant nausea (grade ≥2 according to NCI-CTCAE version 4 up to January 2018, version 5 from January 2018) during the overall phase; complete protection (CP), defined as the sum of CR and no relevant nausea during the overall phase; and need for chemotherapy dose delays and dose reductions due to nausea/vomiting.

Considering the possible unbalanced distribution, the influence of wide within-group variation, and possible interactions, a fixed multivariable regression model was performed to estimate all the effective clinical outcomes according to the type of treatment received (primary versus secondary prophylaxis with NK1-RA for CINV), by using pre-planned adjusting key covariates as stratification factors: sex (male vs. female), age (< vs. ≥70 years old), overweight (BMI < vs. ≥25), underweight (BMI < vs. ≥19), disease spread (early vs. advanced/metastatic), tumor type [esophagogastric cancer vs. the rest, hepatobiliary tumor vs. the rest, colorectal cancer (CRC) vs. the rest], type of NK1-RA used in primary/secondary prophylaxis (NEPA vs. fosaprepitant/aprepitant), concomitant use of opioids drugs (yes vs. no), concomitant use of antidepressant/antipsychotic drugs (yes vs. no), Eastern Cooperative Oncology Group (ECOG) performance status at the start of NK1-RA treatment (0 vs. 1-2), and type of chemotherapy used (doublet vs. triplet) (4–6).

### Statistical analysis

Baseline patients’ characteristics were reported with descriptive statistics and compared among subgroups with the Pearson’s Chi-square test and the Fisher’s exact test, as appropriate. The Chi-square test was also used to compare the effective clinical outcomes across the two subgroups. Logistic regression was used for the multivariate analysis of all the effective clinical outcomes. Odds Ratios (ORs) with 95% confidence intervals (CIs) were calculated using the logistic regression model.

In order to further validate results from the multivariable analysis and reduce biases related to the non-random assignment of the compared strategies (primary vs. secondary CINV prophylaxis with NK1-RA), the propensity score methodology was applied. With this method, the relationship between therapy and outcome is adjusted for the likelihood that a patient has of receiving that treatment, given his baseline characteristics. In detail, the propensity score (chance of receiving primary CINV prophylaxis with NK1-RA) was estimated by a logistic regression model that included, as a dependent variable, the receipt of primary versus secondary CINV prophylaxis with NK1-RA, as covariates, factors that are likely to influence the efficacy of NK1-RA for CINV prophylaxis (sex, age, BMI, disease spread, tumor type, type of NK1-RA used, concomitant use of opioids and/or antidepressant/antipsychotic drugs, ECOG performance status, and type of chemotherapy used). Only subjects with overlapping values of the propensity score were included in the matched groups, with a 1:1 matching between the two study cohorts, allowing a 0.2 caliper (i.e., the maximum tolerated difference in the propensity score between matched subjects). After matching, patients of the two groups had a similar distribution of propensity scores, and, consequently, the two matched groups are similar in terms of sex, age, BMI, disease spread, tumor type, type of NK1-RA used, concomitant use of opioids and/or antidepressant/antipsychotic drugs, ECOG performance status, and type of chemotherapy used, whereas these factors differed greatly between the two unmatched groups. The alpha level for all analyses was set to p < 0.05.

All statistical analyses were performed using MedCalc Statistical Software version 18.11.3 (MedCalc Software bvba, Ostend, Belgium; http://www.medcalc.org; 2019).

## Results

### Patients’ characteristics

**Figure 1:**
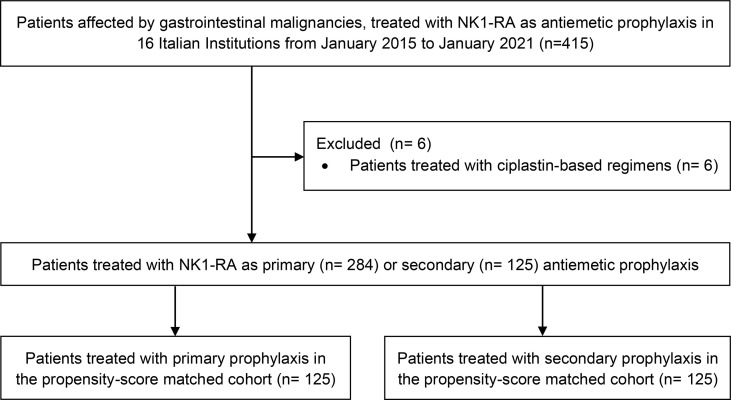
CONSORT diagram with patients selection and disposition.

The CONSORT diagram with patient selection and disposition can be found in **Figure 1**.

In the whole cohort, 409 patients were eligible for analysis. Among them, 284 (69%) and 125 (31%) patients were treated with NK1-RA as primary and secondary antiemetic prophylaxis, respectively.

Patients within the primary prophylaxis cohort were more likely to be treated with aprepitant or fosaprepitant instead of NEPA (58.8% vs. 41.2%, p = 0.031), which, instead, was slightly more adopted as secondary prophylaxis (52.8% vs. 47.2%, p = 0.031). Moreover, 60.6% of patients within the primary prophylaxis cohort received dexamethasone only on day 1, whereas 68.8% of patients in the secondary prophylaxis cohort received dexamethasone on days 1–4 (p < 0.001), concomitantly with NK1-RA.

Triplet combination regimens were more likely to be treated with primary prophylaxis (77.4%), as compared to 57.1% of those who were treated with a doublet combination regimen.

A summary of patients’ characteristics and treatments can be found in **Table 1**.

**Table 1:** Patients’ characteristics in the overall population.

Characteristics, N (%)	All patients N=409	Primary prophylaxis N=284	Secondary prophylaxis N=125	*p*value
**Sex**				
*Male*	216 (52.8)	157 (55.3)	59 (47.2)	
*Female*	193 (47.2)	127 (44.7)	66 (52.8)	0.134
Age, years, median (range)	61 (24-80)	60 (24-80)	61 (24-80)	–
**ECOG PS**				
*0*	240 (58.7)	188 (66.2)	52 (41.6)	
*1*	159 (38.9)	89 (31.3)	70 (56.0)	<0.001
*2*	10 (2.4)	7 (2.5)	3 (2.4)	
**BMI (kg/m2)**				
*Underweight (BMI ≤ 18.5)*	24 (5.9)	19 (6.7)	5 (4.0)	
*Normal weight (BMI 18.5 < BMI ≤ 24.9)*	235 (57.5)	156 (54.9)	79 (63.2)	0.287
*Overweight (25 < BMI ≤ 29.9)*	119 (29.1)	84 (29.6)	35 (28.0)	
*Obese (BMI ≥ 30)*	31 (7.6)	25 (8.8)	6 (4.8)	
**Cancer type**				
*Esophago-gastric*	112 (27.4)	84 (29.6)	28 (22.4)	
*Bilio-pancreatic §*	129 (31.5)	82 (28.9)	47 (37.6)	0.283
*Colon-rectum*	168 (41.1)	118 (41.5)	50 (40.0)	
**Setting of disease**				
*Early*	154 (37.7)	104 (36.6)	50 (40.0)	
*Advanced/metastatic*	255 (62.3)	180 (63.4)	75 (60.0)	0.580
*Liver metastases*	185 (72.5)	130 (72.7)	55 (73.3)	
*Peritoneal metastases*	79 (31.0)	57 (31.7)	22 (29.3)	–
**Previous lines of treatment**				
*None*	328 (80.2)	232 (81.7)	96 (76.8)	0.253
*Yes*	81 (19.8)	52 (18.3)	29 (23.2)	
**Type of NK-1RA**				
Netupitant-Palonosetron (NEPA)	183 (44.7)	117 (41.2)	66 (52.8)	0.031
Aprepitant or Fosaprepitant	226 (55.3)	167 (58.8)	59 (47.2)	
**Prophylaxis with dexamethasone**				
Only on day 1	211 (51.6)	172 (60.6)	39 (31.2)	
On days 1-4	198 (48.4)	112 (39.4)	86 (68.8)	<0.001
**Use of opioids before or during NK1-RA**				
*None*	314 (76.8)	225 (79.2)	89 (71.2)	0.098
*Yes*	95 (23.2)	59 (20.8)	36 (28.8)	
**Use of antidepressants/antipsychotics before orduring NK1-RA**				
*None*	389 (95.1)	272 (95.8)	117 (93.6)	0.332
*Yes*	20 (4.9)	12 (4.2)	8 (6.4)	
**Schedule of treatment**				
*FOLFOX/XELOX/FOLFIRI*	161 (39.4)	92 (32.4)	69 (55.2)	<0.001
*FOLFOXIRI/FOLFIRINOX/FLOT*	248 (60.6)	192 (67.6)	56 (44.8)	
***Baseline chemotherapy dose***				
Standard	300 (73.3)	210 (73.9)	90 (72.0)	
20% or less reduction for at least one drug	83 (20.3)	53 (18.7)	30 (24.0)	0.263
More than 20% reduction for at least one drug	26 (6.4)	21 (7.4)	5 (4.0)	

**Legend:** N, number; ECOG PS, Eastern Cooperative Group Performance Status; BMI, Body Mass Index; NK1-RA, NK1 Receptor Antagonist; § including 2 patients with primary tumour of the duodenum.

### Clinical outcomes analysis

In the whole cohort, protection from acute phase emesis was obtained in 301 (74%) patients, whereas protection from delayed phase emesis was obtained in 329 (80%) patients. Overall protection was obtained in 227 (55%) patients.

The absence of relevant nausea in the overall phase was observed in 278 (68%) patients, whereas rescue antiemetic therapy in the overall phase was needed in 219 (53%) patients. Overall phases CR and CP were observed in 142 (35%) and 135 (33%) patients, respectively.

Finally, chemotherapy delays and dose reductions due to CINV were observed in 50 (12%) and 56 (14%) patients, respectively.

Primary prophylaxis with NK1-RA was not associated with improved protection from acute phase (74% vs. 72%, p = 0.63), delayed phase (82% vs. 77%, p = 0.22), and overall phase (55% vs. 57%, p = 0.75) emesis compared to secondary prophylaxis with NK1-RA.

However, compared to secondary prophylaxis, primary prophylaxis with NK1-RA was associated with less need for rescue therapy (42% vs. 79%, p < 0.000001), a higher rate of CR (43% vs. 16%, p = 0.000008), a lower incidence of relevant nausea (74% vs. 55%, p = 0.0003), and a higher rate of CP (41% vs. 14%, p = 0.000005).

Ultimately, primary prophylaxis with NK1-RA was associated with less chemotherapy dose delays (7% vs. 25%, p = 0.000009) and dose reductions (7% vs. 30%, p = 0.000003) due to nausea or vomiting.

Results of the univariate analysis are shown in **Supplementary Files 2A, B**.

Results of the multivariate analysis are shown in **Tables 2A, B**; primary prophylaxis with NK1-RA was independently associated with a higher protection from emesis during the acute phase (p = 0.04), a lower need for rescue therapy (p < 0.0001), a lower incidence of relevant nausea (p = 0.0005), and a higher rate of CR (p < 0.0001) and CP (p < 0.0001). Finally, chemotherapy dose reductions (p < 0.0001) and delays (p < 0.0001) remained less frequent in patients who received primary prophylaxis with NK1-RA.

**Table 2A:** Multivariate analysis for the effectiveness outcome measures.

		Protection fromemesis (acute phase)		Protection fromemesis (delayed phase)		Protection fromemesis (overall phase)		Absence of relevantnausea (overall phase)	
**Characteristics**	**Allpatients N (%)**	**OR (95% CI)**	***Pvalue***	**OR (95% CI)**	***Pvalue***	**OR (95% CI)**	***Pvalue***	**OR (95% CI)**	***Pvalue***
**Gender**									
*Male*	216 (52.8)	0.56 (0.34-0.93)		1.14 (0.68-1.91)		0.83 (0.55–1.27)		0.94 (0.60-1.46)	
*Female*	193 (47.2)		***<0.05***		*0.62*		*0.39*		*0.77*
**Age (years)**									
*< 75 years*	324 (79.2)	2.22 (1.06–4.65)		1.23 (0.65-2.35)		1.82 (1.05–3.14)		2.02 (1.11-3.70)	
*≥ 75 years*	85 (20.8)		***<0.05***		*0.52*		***<0.05***		***<0.05***
**ECOG PS**									
*0*	240 (58.7)	1.07 (0.61-1.89)		0.55 (0.31-0.96)		0.70 (0.43-1.11)		0.83 (0.05-1.37)	
*1-2*	169 (41.3)		*0.81*		***<0.05***		*0.13*		*0.46*
**Overweight**									
*None*	259 (63.3)	0.83 (0.49-1.40)		1.82 (1.02-3.25)		1.43 (0.92-2.23)		1.43 (0.88-2.32)	
*Yes*	150 (36.7)		*0.49*		***<0.05***		*0.11*		*0.15*
**Underweight**									
*None*	385 (94.1)	0.90 (0.32-2.56)		0.47 (0.19-1.22)		0.88 (0.35-2.17)		0.66 (0.27-1.60)	
*Yes*	24 (5.9)		*0.84*		*0.12*		*0.77*		*0.36*
**Setting of disease**									
*Early*	154 (37.7)	0.42 (0.21-0.84)		2.05 (1.08-3.89)		1.00 (0.58-1.72)		0.95 (0.54-1.68)	
*Advanced/Metastatic*	255 (62.3)		***<0.05***		***<0.05***		*1.00*		*0.86*
**Tumor type - Esophagogastric**									
*None*	297 (72.6)								
*Yes*	112 (27.4)	NE	*1.00*	NE	*1.00*	NE	*1.00*	NE	*1.00*
**Tumor type - Hepatobiliary**									
*None*	281 (68.7)								
*Yes*	128 (31.3)	NE	*1.00*	NE	*1.00*	NE	*1.00*	NE	*1.00*
**Tumor type - Colorectal**									
*None*	241 (58.9)								
*Yes*	168 (41.1)	NE	*1.00*	NE	*1.00*	NE	*1.00*	NE	*1.00*
**Type of prophylaxis**									
*Primary*	284 (69.4)	1.80 (1.01-3.23)		1.20 (0.69-2.09)		0.94 (0.58-1.51)		2.40 (1.46-3.93)	
*Secondary*	125 (30.6)		***<0.05***		*0.53*		*0.79*		***<0.05***
**Type of NK1-RA used**									
*NEPA*	183 (44.7)	3.06 (1.74-5.37)		1.06 (0.61-1.82)		1.43 (0.91-2.23)		1.73 (1.07-2.81)	
*Aprepitant/Fosaprepitant*	226 (55.3)		***<0.05***		*0.84*		*0.12*		***<0.05***
**Concomitant use of opioids**									
*None*	314 (76.8)	1.01 (0.50-2.04)		1.66 (0.79-3.47)		1.43 (0.78-2.62)		1.35 (0.72-2.54)	
*Yes*	95 (23.2)		*0.98*		*1.018*		*0.25*		*0.35*
**Concomitant use ofantidepressant/ antipsychotic drugs**									
*None*	389 (95.1)	0.250 (0.086-0.74)		0.41 (0.15-1.11)		0.24 (0.08-0.73)		0.16 (0.05-0.45)	
*Yes*	20 (4.9)		***<0.05***		***0.08***		***<0.05***		***<0.05***
**Intensity of chemotherapy**									
*Doublet*	161 (39.4)	2.44 (1.34-4.45)		1.03 (0.57-1.87)		1.87 (1.14-3.06)		1.01 (0.60-1.69)	
*Triplet*	248 (60.6)		***<0.05***		*0.92*		***<0.05***		*0.98*

**Legend:** N, number; OR, Odds Ratio; CI, Confidence Interval; ECOG PS, Eastern Cooperative Group Performance Status; NK1-RA NK1 Receptor Antagonist; NEPA, Netupitant/Palonosetron combination; NE, not evaluable/estimable. Bold fond only for statistically significant p-value

**Table 2B:** Multivariate analysis for the effectiveness outcome measures.

		Complete Response(overall phase)		Complete Protection(overall phase)		Chemotherapy dosereductions		Chemotherapy dosedelays	
**Characteristics**	**Allpatients N (%)**	**OR (95% CI)**	***Pvalue***	**OR (95% CI)**	***Pvalue***	**OR (95% CI)**	***Pvalue***	**OR (95% CI)**	***Pvalue***
**Gender**									
*Male*	216 (52.8)	0.74 (0.46-1.19)		0.86 (0.54-1.38)		1.42 (0.76-2.66)		1.09 (0.57-2.09)	
*Female*	193 (47.2)		*0.22*		*0.53*		*0.27*		*0.79*
**Age (years)**									
*< 75 years*	324 (79.2)	1.66 (0.94-2.93)		1.65 (0.93-2.93)		0.88 (0.41-1.90)		0.92 (0.40-2.12)	
*≥ 75 years*	85 (20.8)		*0.08*		*0.08*		*0.74*		*0.84*
**ECOG PS**									
*0*	240	0.51 (0.30-0.87)		0.50 (0.29-0.85)		1.74 (0.87-3.49)		0.48 (0.22-1.03)	
*1-2*	169 (41.3)		***<0.05***		***<0.05***		*0.12*		*0.06*
**Overweight**									
*None*	259 (63.3)	2.03 (1.25-3.30)		2.01 (1.23-3.28)		0.70 (0.35-1.42)		0.86 (0.42-1.75)	
*Yes*	150 (36.7)		***<0.05***		***<0.05***		*0.33*		*0.67*
**Underweight**									
*None*	385 (94.1)	0.93 (0.33-2.65)		1.04 (0.37-2.96)		2.24 (0.69-7.27)		2.27 (0.66-7.80)	
*Yes*	24 (5.9)		*0.90*		*0.94*		*0.18*		*0.19*
**Setting of disease**									
*Early*	154 (37.7)	0.50 (0.28-0.90)		0.48 (0.26-0.88)		0.46 (0.21-1.01)		1.09 (0.49-2.42)	
*Advanced/Metastatic*	255 (62.3)		***<0.05***		***<0.05***		***0.05***		*0.82*
**Tumor type - Esophagogastric**									
*None*	297 (72.6)								
*Yes*	112 (27.4)	NE	*1.00*	NE	*1.00*	NE	*1.00*	NE	*1.00*
**Tumor type - Hepatobiliary**									
*None*	281 (68.7)								
*Yes*	128 (31.3)	NE	*1.00*	NE	*1.00*	NE	*1.00*	NE	*1.00*
**Tumor type - Colorectal**									
*None*	241 (58.9)								
*Yes*	168 (41.1)	NE	*1.00*	NE	*1.00*	NE	*1.00*	NE	*1.00*
**Type of prophylaxis**									
*Primary*	284 (69.4)	5.34 (2.92-9.75)		5.40 (2.91-10.0)		0.14 (0.07-0.29)		0.19 (0.09-0.37)	
*Secondary*	125 (30.6)		***<0.05***		***<0.05***		***<0.05***		***<0.05***
**Type of NK1-RA used**									
*NEPA*	183 (44.7)	1.26 (0.77-2.06)		1.25 (0.76-2.06)		0.98 (0.51-1.88)		1.22 (0.62-2.39)	
*Aprepitant/Fosaprepitant*	226 (55.3)		*0.37*		*0.38*		*0.95*		*0.56*
**Concomitant use of opioids**									
*None*	314 (76.8)	1.09 (0.54-2.18)		0.95 (0.47-1.93)		1.22 (0.51-2.96)		1.50 (0.64-3.55)	
*Yes*	95 (23.2)		*0.81*		*0.88*		*0.65*		*0.35*
**Concomitant use of antidepressant/antipsychotic drugs**									
*None*	389 (95.1)	0.41 (0.10-1.61)		0.27 (0.06-1.32)		0.20 (0.02-1.71		2.58 (0.79-8.45)	
*Yes*	20 (4.9)		*0.20*		*0.11*		*0.14*		*0.12*
**Intensity of chemotherapy**									
*Doublet*	161 (39.4)	3.78 (2.15-6.63)		3.37 (1.92-5.91)		0.73 (0.34-1.55)		1.18 (0.55-2.50)	
*Triplet*	248 (60.6)		***<0.05***		***<0.05***		*0.41*		*0.68*

**Legend:** N, number; OR, Odds Ratio; CI, Confidence Interval; ECOG PS, Eastern Cooperative Group Performance Status; NK1-RA, NK1 Receptor Antagonist; NEPA, Netupitant/Palonosetron combination; NE, not evaluable/estimable. Bold fond only for statistically significant p-value.

Propensity score matching identified, out of the 284 patients in the primary prophylaxis group, 125 patients with the highest propensity score.

After matching for the stratification variables, primary NK1-RA use was still not associated with protection from acute phase (p = 0.34), delayed phase (p = 0.14), and overall phase (p = 0.80) emesis. On the other hand, the absence of relevant nausea was still more frequent in primary NK1-RA users (p = 0.02) and rescue antiemetic therapy was less frequently needed in primary NK1-RA users (p = 0.000007). Furthermore, both overall phases CR (p = 0.00001) and CP (p = 0.00007) were more frequently observed in primary NK1-RA users. Finally, chemotherapy dose delays (p = 0.000009) and reductions (p = 0.0000006) were still less frequently observed in primary NK1-RA users (**Tables 3**).

**Table 3:** Analysis of the effectiveness outcome measures in the propensity score matched population.

Outcome measures	All patients N = 250 (%)	Primary prophylaxis N = 125 (%)	Secondary prophylaxis N = 125 (%)	*P-value*
Protection from emesis (acute phase)	173 (69.2)	83 (66.4)	90 (72.0)	*0.34*
Protection from emesis (delayed phase)	202 (80.8)	106 (84.8)	96 (76.8)	*0.15*
Protection from emesis (overall phase)	139 (55.6)	68 (54.4)	77 (61.6)	*0.79*
Absence of relevant nausea (overall phase)	157 (62.8)	88 (70.4)	69 (55.2)	***<0.05***
Complete Response (overall phase)	72 (28.8)	52 (41.6)	20 (16.0)	***<0.05***
Complete Protection (overall phase)	68 (27.2)	50 (40.0)	18 (14.4)	***<0.05***
Chemotherapy dose reductions	44 (17.6)	7 (5.6)	37 (29.6)	***<0.05***
Chemotherapy dose delays	37 (14.8)	6 (4.8)	31 (24.8)	***<0.05***

N, number. Bold font, only for statistically significant p-value.

## Discussion

Over the past decades, a better comprehension of the multifactorial pathophysiology of CINV, involving interactions between neurotransmitters and receptors in the gastrointestinal tract and central nervous system, led to breakthrough advances in CINV management. However, CINV prevention often remains suboptimal particularly for chemotherapy-induced nausea in the delayed phase and might compromise patients’ adherence to treatments, leading to dose delays or reduction, and ultimately have a negative impact on quality of life (QoL) (6, 17).

According to international guidelines, a 5-HT3-RA plus dexamethasone is recommended for the prevention of acute emesis in MEC-treated patients, but data evaluating the role of dexamethasone or other antiemetics for preventing delayed emesis in MEC are poor.

In this respect, the introduction of NK1-RA agents revolutionized the antiemetic prophylaxis for highly emetogenic chemotherapy (HEC), such as anthracyclines- and cisplatin-based treatments (18, 19), and, among MEC regimens, those including carboplatin (7).

On the other hand, oxaliplatin- and irinotecan-based regimens are still the mainstay of most gastrointestinal malignancies and are associated with an extremely variable risk of nausea and vomiting.

Concerning oxaliplatin, evidence about the use of NK1-RA led to conflicting results (8, 9). While a double-blind trial including 710 patients with CRC receiving oxaliplatin-based treatment and randomized to antiemetic prophylaxis with ondansetron, dexamethasone with or without casopitant, or placebo found no differences in the incidence of vomiting on days 1–5 (11% and 10%, respectively) (8), a different conclusion was reported in a more recent study (9). In this trial including 413 patients with CRC receiving oxaliplatin-based treatment randomized to receive 5HT3-RA and dexamethasone with or without aprepitant, the rate of no vomiting overall and in the delayed phase was higher in the NK1-RA arm (95.7% vs. 83.6% and 95.7% vs. 84.7%, respectively).

On the other hand, evidence concerning the optimal antiemetic management in patients treated with irinotecan-based regimens is even more limited and less consistent.

In a small trial conducted on 44 patients affected by metastatic CRC and treated with irinotecan-based association regimens (including FOLFIRI), antiemetic prophylaxis with dexamethasone and 5HT3-RA resulted in an 86% CR on day 1 and an 82% CR during the delayed phase (20).

More recently, a randomized trial comparing NEPA vs. aprepitant for CINV prevention including 211 patients receiving MEC (more than 65% of whom were treated with oxaliplatin or irinotecan) demonstrated the non-inferiority of NEPA vs. aprepitant in terms of overall CR rate, with a numerically higher trend (64.9% vs. 54.1%) (21).

It is important to underline that none of the abovementioned studies included oxaliplatin- and/or irinotecan-based intensive regimens such as FOLFOXIRI, FOLFIRINOX, and FLOT (or variants), which are commonly used for the treatment of metastatic CRC, pancreatic ductal adenocarcinoma, and gastric cancer (10–16).

With this respect, only a recent small prospective study including 100 patients affected by CRC or pancreatic ductal adenocarcinoma, and treated with FOLFOXIRI or FOLFIRINOX as systemic regimens, found high rates of CR in the overall phase with NEPA for the management of CINV both in antiemetics-naïve patients and in patients previously treated with 5HT3-RA and NK1-RA (22). Concordantly, aprepitant showed a high rate of CR and CP during the first cycle of systemic therapy with FLOT regimen in a cohort of 52 patients (23).

Ultimately, a recent phase III RCT investigating the effect of aprepitant on the prevention of CINV in 248 Chinese women, younger than 50 years, with no or little alcohol use, affected by gastrointestinal malignancies, and treated with FOLFOX or FOLFIRI found a significantly higher rate of CR in the aprepitant group vs. the non-aprepitant group in the overall, acute, and delayed phases as assessed after the first cycle of systemic treatment (24).

Due to the poor and sometimes conflicting evidence, we tried to assess the value of primary prophylaxis compared to secondary prophylaxis for CINV with NK1-RA in patients treated with oxaliplatin- and/or irinotecan-based doublet or triplet regimens in a real-life setting, as far as major effectiveness outcome measures.

Baseline characteristics were fairly balanced, except for the use of dexamethasone as pre- and post-medication for the prevention of both acute and delayed CINV and the rate of patients treated with an intensive triplet systemic regimen, whose risk of CINV was more likely to be prevented with primary prophylaxis with NK1-RA. Furthermore, primary prophylaxis with aprepitant or fosaprepitant was more widely adopted than NEPA.

No substantial differences were observed in terms of acute, delayed, and overall phase emesis between the two groups. Moreover, the rate of emesis in the acute, delayed, and overall phases was substantially in line with the available literature (21–23).

Overall, CR and CP were confirmed as more frequent, and chemotherapy dose reductions and delays as less frequent, in the primary prophylaxis cohort, both in the multivariate analysis and in the propensity score matching population, whose stratification variables included some of the main factors potentially influencing the occurrence of CINV (4, 5).

On the other hand, the low rates of CR and CP might be related to several factors.

Both CR and CP seem to be mainly related to a higher incidence of relevant nausea and, therefore, a higher need for rescue therapy, particularly in the secondary prophylaxis cohort. It is well known that breakthrough nausea still remains a concern and it might be due to causes other than chemotherapy. In this regard, CNS metastases, hyperazotemia, liver metastases, hypercalcemia, gastrointestinal obstruction, or narcotic analgesics might be confounding factors (25). In our study population, about ^2^/_3_ and ^1^/_3_ of patients with metastatic disease were affected by liver or peritoneal metastases, respectively.

At least in part, this might in turn related to a component of anticipatory and breakthrough nausea, which is particularly frequent in patients who previously experienced not well-controlled CINV and might happen even after a more intensive secondary prophylaxis for CINV (25). In turn, this might have led to a higher rate of dose delays or reductions in the secondary compared to the primary prophylaxis cohort.

Including all nausea/emesis events that occurred after 24 h from the administration of chemotherapy might have led to the inclusion of cases of breakthrough nausea/emesis events after 120 h, or of anticipatory emesis.

Moreover, taking into consideration the incidence of a nausea/emesis event during all the cycles of systemic treatment might have further affected the assessment of CR and CP, because the risk of incidence of nausea might particularly increase during multiple cycles of chemotherapy (4).

On the other hand, some patients were not chemotherapy-naïve or treated with opioids or antidepressants during treatment with NK1-RA and this might have further contributed to a worse control of moderate to severe nausea as mentioned above (4).

Another limitation of the study is the wide range of dosage of dexamethasone used. Paradoxically, a higher rate of patients in the secondary prophylaxis cohort adopted a prolonged use of dexamethasone from days 1 to 4. This further underlines the previous considerations. However, to date, there are no data evaluating the role of dexamethasone or other antiemetics in preventing delayed emesis in MEC and this is particularly true for those patients receiving NK1-RA (1).

Of course, an important result is represented by the lower rate of dose reductions and dose delays due to nausea or emesis in the primary compared to the secondary prophylaxis cohort with NK1-RA. This aspect might be particularly relevant in a neoadjuvant/perioperative/adjuvant setting, in which maintaining the dose intensity is important to achieve the best result in terms of oncologic outcomes (26).

The retrospective nature of the present study comes with some ineludible methodological limitations, which are particularly apparent when not-on-purpose data reporting on a subjective issue such as chemotherapy toxicity are involved. This may have entailed potential inter-operator heterogeneity in reporting and inconsistencies in grading nausea, as well as difficulties in assessing the exact timing of a nausea/emesis event, particularly after the first 24 h of each cycle. This aspect, together with unbalanced baseline patients’ characteristics (i.e., higher rate of dexamethasone use only on day 1 in the primary prophylaxis cohort) not included as covariates in the propensity score matching analysis, might have affected some of the effective outcome results, particularly with regard to the interpretation of CR and CP. For all the abovementioned reasons, findings from this study are only hypothesis-generating and should be taken with caution, hoping for prospective and properly designed trials.

## Conclusion

In patients affected by gastrointestinal malignancies, primary prophylaxis of CINV with NK1-RA, 5HT3-RA, and dexamethasone might be appropriate, particularly in those situations at higher risk of moderate to severe nausea or vomiting and in which it is important to avoid chemotherapy dose delays and/or reductions to maintain a proper dose intensity of chemotherapy drugs. It is desirable for the design of “*ad hoc*” clinical trials to assess the net emetogenic risk of different antineoplastic combinations, properly stratifying for known prognostic factors of CINV in these patients.

## Data availability statement

The raw data supporting the conclusions of this article will be made available by the authors, without undue reservation.

## Ethics statement

The studies involving human participants were reviewed and approved by Comitato Etico delle province di L’Aquila e Teramo. The patients/participants provided their written informed consent to participate in this study.

## Author contributions

All authors contributed to the publication according to the ICMJE guidelines for the authorship. All authors read and approved the manuscript and agree to be accountable for all aspects of the research in ensuring that the accuracy or integrity of any part of the work are appropriately investigated and resolved.

## Funding

This study was supported by the Italian Ministry of Health (Ricerca Corrente 2021).

## Conflict of interest

APa reported receiving advisory board fees from GSK, Servier, Pharmamar. RG reported receiving fees from Amgen and Servier and advisory board fees from Amgen, Servier, Bayer and Merck-Serono. LS reported receiving fees from Pierre-Fabre, AstraZeneca, Bayer, Servier, Merck, Amgen. APe reported receiving fees from Eli-Lilly, MSD, BMS, Merck, Servier. BD has received fees from Ipsen, Eisai, Eli Lilly, AstraZeneca, Sanofi, Merck Sharp & Dohme Corp., a subsidiary of Merck & Co., Inc., Kenilworth, NJ, USA, Bayer, Roche, and Amgen; and has received non-financial support from Ipsen, BMS. MG reported receiving fees by Amgen, Merck, Eli-Lilly, Servier, Italfarmaco.

The remaining authors declare that the research was conducted in the absence of any commercial or financial relationships that could be construed as a potential conflict of interest.

## Publisher’s note

All claims expressed in this article are solely those of the authors and do not necessarily represent those of their affiliated organizations, or those of the publisher, the editors and the reviewers. Any product that may be evaluated in this article, or claim that may be made by its manufacturer, is not guaranteed or endorsed by the publisher.
