# Supplementary material for: Primary versus secondary antiemetic prophylaxis with NK1 receptor antagonists in patients affected by gastrointestinal malignancies and treated with a doublet or triplet combination regimen including oxaliplatin and/or irinotecan plus fluoropyrimidines: A propensity score matched analysis
Source: Front Oncol. 2022 Aug 12;12:935826. doi: 10.3389/fonc.2022.935826 (PMC9413268; doi:10.3389/fonc.2022.935826)
Supplement: Supplementary file 1 [file Table_1.docx]

| **Institution** | **Department** |
| --- | --- |
| Clinica Oncologica e Centro Regionale di Genetica Oncologica, Università Politecnica delle Marche, AOU Ospedali Riuniti-Ancona, Italy | Medical Oncology |
| St. Salvatore Hospital, University of L’Aquila, L’Aquila | Medical Oncology |
| Policlinico Universitario Agostino Gemelli, IRCCS - Comprehensive Cancer Center, Rome, Italy | Medical Oncology |
| “Vito Fazzi” Hospital, Lecce, Italy | Medical Oncology |
| S.S. Annunziata Hospital, Chieti, Italy | Clinical Oncology Unit |
| Ospedale del Mare, Naples, Italy | Medical Oncology Unit |
| Città della Salute e della Scienza di Torino, Turin, Italy | Medical Oncology |
| ASL TO4, Ospedale Civile di Ivrea, Ivrea, Turin, Italy | Medical Oncology |
| Unit of Medical Oncology and Biomolecular Therapy, Department of Medical and Surgical Sciences, University of Foggia, Policlinico Riuniti, 71122 Foggia, Italy | Medical Oncology |
| IRCCS Istituto Dermopatico dell'Immacolata (IDI), Rome, Italy | Medical Oncology |
| Fondazione IRCCS Ca' Granda Ospedale Maggiore Policlinico, Milan, Italy | Medical Oncology |
| Santa Chiara Hospital, Trento, Italy | Medical Oncology Unit |
| Santa Maria Goretti Hospital, Latina, Italy | Medical Oncology |
| IRCCS Regina Elena National Cancer Institute, Rome, Italy. | Medical Oncology 1 |
| Medical Oncology Unit A, Policlinico Umberto I, Rome, Italy | Medical Oncology |
| Foundation “Casa Sollievo della Sofferenza IRCCS”, 71013 San Giovanni Rotondo, Italy | Medical Oncology |

**Supplementary file 1.** List of participating centres.
