# Supplementary material for: Primary versus secondary antiemetic prophylaxis with NK1 receptor antagonists in patients affected by gastrointestinal malignancies and treated with a doublet or triplet combination regimen including oxaliplatin and/or irinotecan plus fluoropyrimidines: A propensity score matched analysis
Source: Front Oncol. 2022 Aug 12;12:935826. doi: 10.3389/fonc.2022.935826 (PMC9413268; doi:10.3389/fonc.2022.935826)
Supplement: Supplementary file 2 [file Table_2.docx]

| ***Supplementary file 2A. Univariate analysis of the effectiveness outcome measures*** | | | | | | | | | |
| --- | --- | --- | --- | --- | --- | --- | --- | --- | --- |
| **Characteristics** | **All patients**  **N (%)** | **Protection from emesis**  **(acute phase)**  **N (%)** | ***p* value** | **Protection from emesis**  **(delayed phase)**  **N (%)** | ***p* value** | **Protection from emesis**  **(overall phase)**  **N (%)** | ***p* value** | **Absence of relevant nausea**  **(overall phase)**  **N (%)** | ***p* value** |
| **Gender** |  | | | | | | | | |
| ***Male*** | 216 (52.8) | 167 (77.3) | *0.07* | 174 (80.5) | *1.00* | 125 (57.8) | *0.30* | 152 (70.3) | *0.28* |
| ***Female*** | 193 (47.2) | 134 (69.4) |  | 155 (80.3) |  | 102 (52.8) |  | 126 (65.3) |  |
| **Age (years)** |  | | | | | | | | |
| ***< 75 years*** | 324 (79.2) | 227 (70.1) | ***<0.05*** | 260 (80.2) | *1.00* | 169 (52.1) | ***<0.05*** | 212 (65.4) | ***<0.05*** |
| ***≥ 75 years*** | 85 (20.8) | 74 (87.1) |  | 69 (81.1) |  | 58 (68.2) |  | 66 (77.6) |  |
| **ECOG PS** |  | | | | | | | | |
| ***0*** | 240 (58.7) | 174 (72.5) | *0.57* | 201 (83.8) | *0.06* | 136 (56.7) | *0.61* | 172 (71.7) | *0.07* |
| ***1-2*** | 169 (41.3) | 127 (75.1) |  | 128 (75.7) |  | 91 (53.8) |  | 106 (62.7) |  |
| **Overweight** |  | | | | | | | | |
| ***None*** | 259 (63.3) | 193 (74.5) | *0.64* | 199 (76.8) | ***<0.05*** | 137 (52.9) | *0.18* | 167 (64.5) | ***<0.05*** |
| ***Yes*** | 150 (36.7) | 108 (72.0) |  | 130 (86.7) |  | 90 (60.0) |  | 111 (74.0) |  |
| **Underweight** |  | | | | | | | | |
| ***None*** | 385 (94.1) | 284 (73.8) | *0.81* | 313 (81.3) | *0.11* | 215 (55.8) | *0.67* | 264 (68.6) | *0.37* |
| ***Yes*** | 24 (5.9) | 17 (70.8) |  | 16 (66.7) |  | 12 (50.0) |  | 14 (58.3) |  |
| **Setting of disease** |  | | | | | | | | |
| ***Early*** | 154 (37.7) | 132 (85.7) | ***<0.05*** | 116 (75.3) | ***0.05*** | 93 (60.4) | *0.13* | 106 (68.8) | *0.83* |
| ***Advanced/Metastatic*** | 255 (62.3) | 169 (66.3) |  | 213 (83.5) |  | 134 (52.5) |  | 172 (67.5) |  |
| **Tumor type - Esophagogastric** |  | | | | | | | | |
| ***None*** | 297 (72.6) | 204 (68.7) | ***<0.05*** | 238 (80.1) | *0.89* | 149 (50.2) | ***<0.05*** | 199 (67.0) | *0.55* |
| ***Yes*** | 112 (27.4) | 97 (86.6) |  | 91 (81.3) |  | 78 (69.6) |  | 79 (70.5) |  |
| **Tumor type - Hepatobiliary** |  | | | | | | | | |
| ***None*** | 281 (68.7) | 205 (73.0) | *0.72* | 230 (81.9) | *0.29* | 160 (56.9) | *0.39* | 198 (70.5) | *0.11* |
| ***Yes*** | 128 (31.3) | 96 (75.0) |  | 99 (77.3) |  | 67 (52.3) |  | 80 (62.5) |  |
| **Tumor type - Colorectal** |  | | | | | | | | |
| ***None*** | 241 (58.9) | 193 (80.1) | ***<0.05*** | 191 (79.3) | *0.53* | 146 (60.6) | ***<0.05*** | 159 (66.0) | *0.33* |
| ***Yes*** | 168 (41.1) | 108 (64.3) |  | 138 (82.1) |  | 81 (48.2) |  | 119 (70.8) |  |
| **Type of prophylaxis** |  | | | | | | | | |
| ***Primary*** | 284 (69.4) | 211 (74.3) | *0.63* | 233 (82.0) | *0.23* | 156 (54.9) | *0.75* | 209 (73.6) | ***<0.05*** |
| ***Secondary*** | 125 (30.6) | 90 (72.0) |  | 96 (76.8) |  | 71 (56.8) |  | 69 (55.2) |  |
| **Type of NK1-RA used** |  | | | | | | | | |
| ***NEPA*** | 183 (44.7) | 154 (84.2) | ***<0.05*** | 146 (79.8) | *0.80* | 110 (60.1) | *0.11* | 131 (71.6) | *0.17* |
| ***Aprepitant/Fosaprepitant*** | 226 (55.3) | 147 (65.0) |  | 183 (81.0) |  | 117 (51.8) |  | 147 (65.0) |  |
| **Concomitant use of opioids** |  | | | | | | | | |
| ***None*** | 314 (76.8) | 234 (74.5) | *0.43* | 251 (79.9) | *0.77* | 174 (55.4) | *1.00* | 218 (69.4) | *0.26* |
| ***Yes*** | 95 (23.2) | 67 (70.5) |  | 78 (82.1) |  | 53 (55.8) |  | 60 (63.2) |  |
| **Concomitant use of antidepressant/**  **antipsychotic drugs** |  | | | | | | | | |
| ***None*** | 389 (95.1) | 291 (74.8) | ***<0.05*** | 316 (81.2) | *0.08* | 222 (57.1) | ***<0.05*** | 272 (69.9) | ***<0.05*** |
| ***Yes*** | 20 (4.9) | 10 (50.0) |  | 13 (65.0) |  | 5 (25.0) |  | 6 (30.0) |  |
| **Intensity of chemotherapy** |  | | | | | | | | |
| ***Doublet*** | 161 (39.4) | 130 (80.7) | ***<0.05*** | 131 (81.4) | *0.80* | 102 (63.4) | ***<0.05*** | 109 (67.7) | *1.00* |
| ***Triplet*** | 248 (60.6) | 171 (69.0) |  | 198 (79.8) |  | 125 (50.4) |  | 169 (68.1) |  |

***Legend:*** N, number; CI, Confidence Interval; ECOG PS, Eastern Cooperative Group Performance Status; NK1-RA NK1 Receptor Antagonist; NEPA, Netupitant/Palonosetron combination. Bold font only for statistically significant p-values.

| ***Supplementary file 2B. Univariate analysis of the effectiveness outcome measures (continued)*** | | | | | | | | | |
| --- | --- | --- | --- | --- | --- | --- | --- | --- | --- |
| **Characteristics** | **All patients**  **N (%)** | **Complete Response (overall phase)**  **N (%)** | ***p* value** | **Complete Protection (overall phase)**  **N (%)** | ***p* value** | **CHT dose reductions N (%)** | ***p* value** | **CHT dose delays**  **N (%)** | ***p* value** |
| **Gender** |  | | | | | | | | |
| ***Male*** | 216 (52.8) | 82 (37.9) | *0.15* | 76 (35.1) | *0.34* | 25 (11.6) | *0.20* | 24 (11.1) | *0.55* |
| ***Female*** | 193 (47.2) | 60 (31.1) |  | 59 (30.6) |  | 31 (16.1) |  | 26 (13.4) |  |
| **Age (years)** |  | | | | | | | | |
| ***< 75 years*** | 324 (79.2) | 104 (32.1) | ***<0.05*** | 99 (30.6) | ***0.05*** | 44 (13.6) | *0.86* | 41(12.7) | *0.71* |
| ***≥ 75 years*** | 85 (20.8) | 38 (44.7) |  | 36 (42.4) |  | 12 (14.1) |  | 9 (10.6) |  |
| **ECOG PS** |  | | | | | | | | |
| ***0*** | 240 (58.7) | 98 (40.8) | ***<0.05*** | 95 (39.6) | ***<0.05*** | 23 (9.6) | ***<0.05*** | 28 (11.7) | *0.76* |
| ***1-2*** | 169 (41.3) | 44 (26.0) |  | 40 (23.7) |  | 33 (19.5) |  | 22 (13.0) |  |
| **Overweight** |  | | | | | | | | |
| ***None*** | 259 (63.3) | 77 (29.7) | ***<0.05*** | 73 (28.2) | ***<0.05*** | 41( 15.8) | *0.10* | 36 (13.9) | *0.21* |
| ***Yes*** | 150(36.7) | 65 (43.3) |  | 62 (41.3) |  | 15 (10) |  | 14 (9.3) |  |
| **Underweight** |  | | | | | | | | |
| ***None*** | 385 (94.1) | 135 (35.1) | *0.66* | 128(33.2) | *0.82* | 51( 13.2) | *0.35* | 46 (0.12) | *0.52* |
| ***Yes*** | 24 (5.9) | 7 (29.2) |  | 7 (29.2) |  | 5 (20.8) |  | 4 (16.6) |  |
| **Setting of disease** |  | | | | | | | | |
| ***Early*** | 154 (37.7) | 66 (42.9) | ***<0.05*** | 64 (0.42) | ***<0.05*** | 29 (18.8) | ***<0.05*** | 18 (11.7) | *0.88* |
| ***Advanced/Metastatic*** | 255 (62.3) | 76 ( 29.8) |  | 71 (27.8) |  | 27 (10.6) |  | 32 (12.5) |  |
| **Tumor type - Esophagogastric** |  | | | | | | | | |
| ***None*** | 297 (72.6) | 88 (29.7) | ***<0.05*** | 84 (28.3) | ***<0.05*** | 36 (12.1) | *0.15* | 41 (13.8) | *0.13* |
| ***Yes*** | 112 (27.4) | 54 (48.2) |  | 51 (45.5) |  | 20 (17.9) |  | 9 (12.4) |  |
| **Tumor type - Hepatobiliary** |  | | | | | | | | |
| ***None*** | 281 (68.7) | 106 (37.7) | *0.07* | 101 (35.9) | *0.07* | 38 (13.5) | *0.88* | 27 (9.6) | ***<0.05*** |
| ***Yes*** | 128 (31.3) | 36 (28.1) |  | 35 (27.3) |  | 18 (14,1) |  | 23 (18.0) |  |
| **Tumor type - Colorectal** |  | | | | | | | | |
| ***None*** | 241 (58.9) | 90 (37.3) | *0.21* | 85 (35.3) | *0.29* | 39 ( 16.2) | *0.08* | 33(13.7) | *0.36* |
| ***Yes*** | 168 (41.1) | 52 (31.0) |  | 50 (29.8) |  | 17 (10.1) |  | 17 (10.1) |  |
| **Type of prophylaxis** |  | | | | | | | | |
| ***Primary*** | 284 (69.4) | 122 (43.0) | ***<0.05*** | 117 (41.2) | ***<0.05*** | 19 (6.7) | ***<0.05*** | 19 (6.7) | ***<0.05*** |
| ***Secondary*** | 125 (30.6) | 20 (16.0) |  | 18 (14.4) |  | 37 (29.6) |  | 31 (24.8) |  |
| **Type of NK1-RA used** |  | | | | | | | | |
| ***NEPA*** | 183 (44.7) | 69 (37.7) | *0.30* | 65 (35.5) | *0.34* | 26 (14.2) | *0.89* | 27 (14.8) | *0.17* |
| ***Aprepitant/Fosaprepitant*** | 226 (55.3) | 73 (32.3) |  | 70 (31.3) |  | 30 (13.3) |  | 23 (10.2) |  |
| **Concomitant use of opioids** |  | | | | | | | | |
| ***None*** | 314 (76.8) | 117 (37.3) | ***0.05*** | 113(51.4) | ***<0.05*** | 40 (12.7) | *0.31* | 32 (10.2) | ***<0.05*** |
| ***Yes*** | 95 (23.2) | 25 (26.3) |  | 22 (23.2) |  | 16 (16.8) |  | 18 (18.9) |  |
| **Concomitant use of antidepressant/**  **antipsychotic drugs** |  | | | | | | | | |
| ***None*** | 389 (95.1) | 139 (35.7) | *0.09* | 133 (32.2) | ***<0.05*** | 55 (14.2) | *0.33* | 45 (11.6) | *0.08* |
| ***Yes*** | 20 (4.9) | 3 (15.0) |  | 2 (10.0) |  | 1 (5.0) |  | 5 (25.0) |  |
| **Intensity of chemotherapy** |  | | | | | | | | |
| ***Doublet*** | 161 (39.4) | 67 (41.6) | ***<0.05*** | 62 (38.5) | *0.07* | 24 (14.9) | *0.56* | 23 (14.3) | *0.35* |
| ***Triplet*** | 248 (60.6) | 75 (30.2) |  | 73 (29.4) |  | 32 (12.9) |  | 27 (10.8) |  |

***Legend:*** N, number; CHT, chemotherapy; ECOG PS, Eastern Cooperative Group Performance Status; NK1-RA NK1 Receptor Antagonist; NEPA, Netupitant/Palonosetron combination. Bold font only for statistically significant p-values.
